# Can Physicochemical Properties Alter the Potency of Aeroallergens? Part 2 – Impact of Physicochemical Properties

**DOI:** 10.1007/s11882-024-01173-7

**Published:** 2024-09-20

**Authors:** Carla S. S. Teixeira, Bruno Carriço-Sá, Caterina Villa, Isabel Mafra, Joana Costa

**Affiliations:** REQUIMTE-LAQV, Faculdade de Farmácia, Universidade do Porto, Rua de Jorge Viterbo Ferreira, 228, 4050-313 Porto, Portugal

**Keywords:** Aeroallergens, Physicochemical properties, Protein allergenicity, Abundance, Protein structure, Aggregation, Ligand/lipid-binding

## Abstract

**Purpose of Review:**

A holistic perspective on how physicochemical properties modulate the allergenicity of proteins has recently been performed for food allergens, launching the challenge of a similar analysis for aeroallergens. After a first review on aeroallergen classification into protein families (Part 1), this second part (Part 2) will exploit the impact of physicochemical properties (abundance/biological function, protein structure/presence of post-translational modifications, ligand/cofactor/lipid-binding) on inhalant protein allergenicity.

**Recent Findings:**

The abundance linked to biological function is correlated with increased allergenic risk for most protein families, while the loss of structural integrity with consequent destruction of conformational epitopes is well linked with decreased allergenicity. Ligand-binding effect totally depends on the ligand type being highly variable among aeroallergens.

**Summary:**

Knowledge about the physicochemical properties of aeroallergens is still scarce, which highlights the need for research using integrated approaches (in silico and experimental) to generate and analyze new data on known/new aeroallergens.

## Introduction

For several years, the question “what makes a protein into an allergen?” has fomented the study of the causative agents of allergies [1]. The reasons beyond the fact that some people are allergic to specific proteins and not to others and why a significant portion of the global population suffers from allergies are still unanswered questions [2, 3]. Recent literature reviews provide a holistic overview of how physicochemical properties shape protein allergenicity in terms of food allergies. On this topic, some physicochemical properties have been advanced as having more impact on protein allergenicity, considering food allergens of specific protein families [2, 3]. Some physicochemical properties seem to exhibit a similar influence in food allergens, independently of their origin (animal versus plant), such as the case of abundance linked to biological function, stability towards light/radiation, resistance to intestinal proteolysis (trypsin/chymotrypsin), and lipid interactions during gastrointestinal digestion. All the referred properties have a protective effect on protein allergenicity as they contribute to maintaining or increasing their allergenic potency. Changes in protein structure and in protein integrity seem to have a high impact on the IgE-binding capacity of food allergens, as they contribute to reducing/mitigating their allergenicity, especially when proteins lose their primary structure due to fragmentation (chemical/enzymatic hydrolysis) [2, 3]. On the other hand, properties such as post-translational modifications (PTM), glycation (Maillard reaction), or aggregation phenomena show many contradictory effects among protein families or even within members of the same protein family, precluding to estimate their actual role in protein allergenicity [2, 3].

Respiratory allergies (allergic rhinitis, allergic asthma, allergic sinusitis, occupational allergy) have major constraints on the quality of life of allergic individuals, as they affect almost half of the world’s population [4, 5]. In this sense, it is important to identify and describe not only the main culprits causing these allergies, as performed in Part 1 of this review, but also to assess which physicochemical characteristics impact their allergenicity, being the focus of the present work (Part 2).

For the aeroallergens, the influence of distinct physicochemical properties on protein allergenicity, focusing on families has been described herein for the first time. With the objective of bringing new insights into the causative agents of respiratory allergies, this Part 2 review will be specifically focused on assessing the role of several physicochemical properties shaping their allergenicity. Contrarily to food allergens, exposure to aeroallergens is environmental, meaning that the physicochemical properties of aeroallergens are slightly different from food allergens. In general, aeroallergens are not submitted to processing techniques, although in some cases, they might result from them, such as the case of respiratory allergies triggered by flours/grains or cooking vapors [6]. The aeroallergens are also unaffected by complex matrices, as they are very small aero-transported particles. On the other hand, there are several environmental and geographical factors, such as season, temperature, radiation, pollution, amount, and typology of vegetation, among others, that are known to affect some of the physicochemical properties of aeroallergens.

This Part 2 review on aeroallergens intends to provide a novel holistic and harmonized overview of how diverse physicochemical properties affect inhalant protein allergenicity, namely their abundance and widespread availability, their structure and function, as well as the occurrence of PTM, ligand/lipid-binding or other interactions.

## Can Physicochemical Properties Modulate Allergenicity?

The information on how the physicochemical properties of aeroallergens may impact protein allergenicity is still quite dispersed and full of gaps, making it extremely difficult to outline a holistic perspective on such an important topic. Nevertheless, the most relevant physicochemical properties have been evaluated for different aeroallergens, being gathered and critically analyzed in the following subsections. The methodology and terminology (allergenicity, IgE-binding capacity) employed for the evaluation of the impact of different physicochemical properties on the allergenicity of inhalant proteins was based on data compilation from different assays following the same strategy as for the analysis of plant and animal food allergens [2, 3].

### Abundance

In general, the expression of high quantities of pollens is greatly associated with an increased possibility of triggering respiratory allergic reactions in pollen-sensitized individuals. The increase in global temperature seems to contribute to aggravate this problem, leading to a more drastic clinical expression of common allergic diseases (allergic asthma, allergic rhinitis) [7]. In addition, temperature rise also contributes to earlier plant pollination, representing an increased risk for overlapping different pollen seasons [7]. Along with pollen-augmented risk exposure, the temperature rise also stimulates the release of fungal spores, thus contributing to intersecting patterns of aeroallergens [7, 8]. Most pollen and fungal spores are composed of several aeroallergens, all belonging to different families, meaning that their increased expression in respective sources can be positively correlated with their enhanced potential for eliciting an allergic reaction in respiratory-allergic individuals, which seems to be the case of nonspecific lipid transfer protein (nsLTP), subtilases, β-expansins (group 1 grass pollens), and pathogenesis-related-10 (PR-10) proteins. The EF-hand and tropomyosin aeroallergens are regulatory proteins less abundant in insects/mites/fungi than the aeroallergens associated with pollen germination or plant defense. However, these minor aeroallergens are known to be very relevant due to their high potential to trigger severe allergic reactions.

Profilins are greatly expressed near the pollinic tube, making them highly abundant in pollen [9], although it is not clear how abundance impacts profilin allergenicity. nsLTP are tissue-specific since they are primarily found in the epidermal tissues of aerial organs, such as leaves, fruits, and pollens [10]. In the case of aeroallergens, their high expression and location in pollen make them important inhalant sensitizers with significant clinical relevance, especially in LTP-rich areas (e.g., Ole e 7, olive tree) [11]. The expression of α-amylases is highly dependent on the species, but their association with important occupational allergies seems to be correlated with enhanced allergenic risk [12]. Allergenic subtilases are typically abundant and stable proteins in their respective allergenic sources [13, 14]. Owing to their extensive secretion by fungi and/or presence in cockroach frasses and mite fecal pellets, they can be easily airborne transported. Jointly, these properties could justify, at least partially, their classification as major aeroallergens, with IgE-binding frequencies higher than 50% [15, 16]. Group 1 allergens (β-expansins) are abundantly (up to 4% of total protein) and specifically expressed in grass pollen, being responsible for inducing hay fever and seasonal asthma in around 200–400 million humans [17, 18]. Contrarily to this subset, other β-expansins are generally found in very low abundance and tightly bound to the cell wall but still maintaining their allergenicity [19].

PR-10 proteins have been found in various plant species, particularly in reproductive tissues like pollen, seeds, and fruits. Their abundant expression can be developmentally regulated or induced by various stress factors, including biotic (infection by bacteria, fungi, parasites, insects, and weeds) and abiotic factors (heat, cold, drought, and salinity). PR-10 proteins can present different abundances depending on the plant species and the conditions of pollen collection, representing on average 22% of the water-soluble protein fraction [20, 21]. Independently on the plant species, the PR-10 proteins (Bet v 1 family) reveal high IgE-binding capacity with the reactive residues located at epitopic regions, which are greatly preserved [21]. In general, the abundance is better linked to the biological function of each protein family, suggesting a stronger correlation between abundance/biological function and the increased potential for respiratory allergy triggers, as it has also been described for other allergen families [2, 3].

Main findings (Table 1):

- The high abundance of nsLTP, subtilases, α-amylases, β-expansins (group 1 grass pollens), and PR-10 proteins is correlated with an increased risk of respiratory allergic reactions.
- The abundance of nsLTP and their tissue location is related to enhanced allergic elicitation risk.
- The relatively low abundance of EF-hand proteins and tropomyosins does not compromise their tendency to an increased allergenic risk.
- The correlation of a high abundance of profilins and increased allergenic risk is still unclear.

**Table 1:** Main conclusions about the influence of each physicochemical property on the protein IgE-binding capacity

	Impact on IgE-binding capacity	Supporting evidence/Main concerns
Abundance	Low	Low or high abundant proteins are potent aeroallergens (e.g. EF-hand proteins—low abundant, β-expansins—high abundant)
Biological function	High	Potent aeroallergens display biological functions on regulation, transport, enzymatic activity and defense
Protein structure		
Loss of 3D	Low	Contradictory effects. Loss of structural stability contribute to maintain/increase (unmasking hidden linear epitopes) (e.g. nsLTP, subtilases) or decrease the IgE-binding capacity (destruction of conformational epitopes) (e.g. lipocalins, profilins)
Loss of S–S bonds	Low	Contradictory effects. Loss of structural stability contribute to maintain/increase (unmasking hidden linear epitopes) (e.g. nsLTP) or decrease the IgE-binding capacity (destruction of conformational epitopes) (e.g. lipocalins)
Aggregation	High	Formation of aggregates leading to increased number of conformational epitopes contribute to maintain/increase the IgE-binding capacity of polcalcins, lipocalins, Ole e 1-like proteins and PR-10
PTM		
Glycosylation	Low	Contradictory effects are found for aeroallergens, either no effect or increased IgE-binding capacity. Information limited to calycin (lipocalins), subtilases, and Ole e 1-like proteins
Phosphorylation	Limited	Phosphorylation described for monomeric profilins but no correlation with their IgE-binding capacity. Information limited to profilins
Ligand/cofactor binding	High	The presence of ligands/cofactors contribute to maintain or increase the IgE-binding capacity for EF-hand proteins (polcalcins, troponin C), profilins, calycin (lipocalins) and PR-10 proteins
Lipid-binding	Limited/high	Lipid-binding contributes to maintain the IgE-binding capacity, but information limited to EF-hand (FABP) and nsLTP

*FABP* fatty acid-binding proteins, *IgE* immunoglobulin E, *nsLTP* nonspecific lipid transfer protein, *PR10 proteins* pathogenesis related-10 proteins, *PTM* post-translational modifications

### Protein Structure and Aggregation Phenomenon

In general, aeroallergens are mostly acidic proteins (82% acidic, with an average pI of 5.7), being notably distinct from non-allergens (20% acidic, with an average pI of 7.6), but similar to food allergens (88% acidic, pI ~ 5.8). In a neutral aqueous solution, allergens have a negative charge, while non-allergens are neutral to positively charged [22]. Allergens have an overall trend for holding a negative electrostatic potential, compared to non-allergens that exhibit positive electrostatic potentials (83% of aeroallergens and 72% of food allergens versus 40% for non-allergens, p < 0.0001). Interestingly, a few allergens (< 10%) are known to possess positive charge and neutral/basic pH, but the opposite is not true. Nevertheless, these properties do not account with potential PTM, meaning that some variability may be expected when evaluating new aeroallergens [22].

As EF-hand members, polcalcins Bet v 4 and Phl p 7 are known to undergo oligomerization, with transitions from monomers to dimers/oligomers being favored by increasing temperature or low concentrations of destabilizing agents (e.g., sodium dodecyl sulfate - SDS) [23]. The alteration in the quaternary structure of these proteins (molecular metamorphosis) is prompted and controlled by a combination of EF-hand rearrangements and domain transaction. In both cases, the uncommon oligomerization behavior proposes a direct rationalization of how allergens can undertake the crosslinking of IgE on mast cells, which is a characteristic of allergens [23]. Plant recombinant Che a 3 (polcalcin) presents high IgE-reactivity, as well as high basophil degranulation-triggering capacity compared to Che a 3 peptide, as the latter has disrupted 3D structure [24]. Regarding troponin C, it is estimated that the rupture of its conformational structure contributes to the loss of IgE-binding capacity. This was evidenced by the loss of the immunodominant conformational IgE epitope on the C-terminal region of Der p 39 upon alteration of the 3D structure [25].

Profilins are proteins with compact globular structures, and the loss of their 3D structures seems to contribute to a decrease in their IgE-binding capacity [26, 27]. Aeroallergenic lipocalins share a conserved 3D structure with a high tendency for oligomerization and complexation with soluble macromolecules, being prone to form oligomers (dimers and tetramers) [28, 29]. Allergenic lipocalins form transient dimers in solution, while hypoallergenic counterparts exist only in monomeric structures, highlighting the importance of di/oligomerization as a necessary feature for lipocalin allergenicity [30]. Oligomerization of an allergenic molecule might facilitate the IgE crosslinking on immune cells by multiplying identical epitopes [29]. Most aeroallergenic nsLTP have a highly preserved 3D structure that is stabilized by 4-disulfide bridges. The loss of their 3D structure does not affect their allergenicity, thus maintain their allergenic potency both in terms of aeroallergen and food allergen, as is the case of Pru p 3 [31, 32].

For subtilisin-like serine proteases, limited information was found on their structural conformation. An immunodominant linear IgE epitope has been described for Pen c 13 (subtilase) located at residues 261–274, with Gly^270^ and Lys^274^, being directly recognized by human IgE. As these residues are positioned on a loop-like structure at/or near the surface of Pen c 13 [33], they are expected to maintain their ability to bind the IgE, even when the 3D conformation is altered. β-Expansins evidence a conserved 3D structure, common to most members, but the effect of protein structural alteration on their allergenicity is still quite unknown. The Ole e 1-like proteins seem to show a high degree of structural similarity, but the Ole e 1-like loop regions might comprise IgE-binding sites that are quite distinct for each allergen [34]. Additionally, Ole e 1 subunits form dimers with higher allergenicity than their monomeric structures [35]. However, this phenomenon was not reported/tested for other Ole e 1-like proteins. PR-10 allergens are described as small proteins that are present as a mixture of monomers, dimers, and oligomers, with dimerization being crucial for IgE crosslinking on mast cells. Therefore, the destruction of the dimers or high-order oligomers seems to decrease the IgE-binding capacity of these proteins [36]. The loss of structural integrity of tropomyosins seems to have a minor impact on their IgE-binding capacity as these proteins possess different sequential epitopes that maintain their reactivity upon protein unfolding [37], being also a common trace of allergenic food tropomyosins [2].

Main findings (Table 1):

- Polcalcins, profilins, lipocalins, subtilases, Ole e 1-like proteins, PR-10, and tropomyosins tend to form aggregated forms, ranging from dimers to oligomers, which contribute to maintaining or increasing the IgE-binding capacity of polcalcins, lipocalins, Ole e 1- like proteins and PR-10. For the remaining families, no correlation could be found.
- The IgE-binding capacity of EF-hand proteins (polcalcins and troponin C), profilins, lipocalins, and PR-10 proteins seems to be reduced by the loss of their quaternary conformations or oligomeric structures (destruction of conformational epitopes).
- The IgE-binding capacity of nsLTP, subtilisin-like serine proteases, and tropomyosins seems to be maintained even after the loss of native structural integrity (presence of linear epitopes).
- The IgE-binding capacity of Ole e 1-like proteins upon structural alteration seems to be species-dependent, as their members might not share the same epitopic regions.
- The IgE-binding capacity of β-expansins upon destruction of their structural integrity was not yet reported.

### Post-Translational Modifications

PTM are known to present some impact on protein allergenicity, although they are fully dependent on the type of modification [2, 3]. The most commonly reported is glycosylation, although other PTM can also occur in allergenic proteins [2, 3].

Aeroallergens from the EF-hand family do not present any identified PTM. Profilins and nsLTP are panallergens, which are often associated with cross-reactivity and co-sensitization phenomena, as well as the occurrence of various syndromes. There are also no reports of PTM in aeroallergenic nsLTP [38], but profilins seem to be potentially phosphorylated in their monomeric forms (not in dimers/tetramers) [39]. Within the calycin superfamily, lipocalins can be N- and/or O-glycosylated or non-glycosylated, presenting one or more intramolecular disulfide bonds that stabilize the structure. They are also thought to be phosphorylated. As few lipocalins are glycosylated, this PTM does not seem to be correlated with their allergenic capacity [29]. As a proof of concept, Bla g 4 was found to be glycosylated, and its deglycosylation with endoglycosidase F did not alter its IgE-reactivity, meaning that glycosylation did not affect the IgE-binding capacity of the native protein [40]. Regarding cytosolic fatty acid-binding proteins (FABP), no PTM (glycosylation) has been reported [41]. A critical characteristic in plant subtilases is glycosylation, a PTM that regulates their activity [42, 43], although their role in allergenicity is still unknown. Some β-expansins seem to be glycosylated (e.g., Ory s 1, Lol p 1) [44, 45], though their correlation with protein allergenicity has not been described. Natural Ole e 1-like proteins isolated from pollen exhibit partial N-glycosylation, which might be associated with their IgE-binding capacity [46]. Ole e 1 exhibits a high degree of polymorphism in protein sequence and attached oligosaccharide structures, suggesting that glycosylation is well-correlated with increased allergenicity [47, 48]. As food allergens, few tropomyosins were found to be glycosylated or acetylated, but regarding aeroallergens, no PTM was reported in tropomyosins [45, 49].

Main findings (Table 1):

- No PTM was found in proteins of EF-hand, α-amylases, nsLTP, FABP, PR-10 and tropomyosin families.
- Glycosylation occurs in calycin (lipocalins), subtilases, and Ole e 1-like proteins. In lipocalins, glycosylation does not seem to affect the allergenicity, while in Ole e 1-like proteins, it increases allergenicity. For subtilases, glycosylation has no known role in protein allergenicity.
- Although phosphorylation was described for calycin (lipocalins) and profilins in their monomeric form, no correlation with their allergenicity has been established so far.

### Ligand/Cofactor-Binding

In general, ligands can increase the stability of allergens towards thermal and/or proteolytic degradation, although their effects are also case-dependent. Ligands can function as immunomodulatory agents, unbalancing Th1/Th2 towards Th2 polarization. Ligand‐binding allergens are known to expose the immunological system to a diversity of biologically active compounds, but their influence on the induction process is still not well understood [50].

The IgE recognition of EF-hand proteins has been associated with their ligand-binding capacity, namely to divalent cations. This family of proteins predominantly binds Ca^2+^, but members can also bind Mg^2+^, though with less affinity and efficiency [51, 52]. Polcalcins have been described as depending on the calcium-binding to be functional since the use of chelating agents or the mutation of the calcium-coordinating residues disturbs the conformational integrity of EF-hand allergens. This suggests a prominent role of calcium in the IgE recognition of polcalcins, whose absence seems to destroy protein integrity and conformational epitopes (feature associated with other important inhalant allergens), thus strongly reducing protein allergenicity (e.g., Phl p 7) [53, 54]. Based on this physicochemical property, some hypoallergenic proteins were produced and incorporated into vaccines for allergy treatment [55]. Additionally, the carboxyl-terminal calcium-binding domain is determinant to the IgE-reactivity of Bet v 4 among pollen-allergic individuals [52].

In line with polcalcins, the aeroallergenic troponins C also coordinate ligands such as calcium and their IgE-binding capacity depends on calcium-binding [56–58]. Profilins can bind to the proline-rich regions of proteins (poly(l-proline)), like formin-related proteins and vasodilator-stimulated phosphoprotein, as well as interact with membrane-located phospholipids, such as phosphatidylinositol 4,5-bisphosphate and phosphatidylinositol 4-phosphate, and cytoskeletal components [59]. Their oligomeric structure often possesses weak affinity to poly(l-proline), most likely owing to the unavailability of binding sites in dimers and tetramers [39]. The most common IgE-reactive epitopes in profilins seem to be placed in regions with preserved sequence/secondary structure and overlapping the ligand domains, thus demonstrating that the native ligand-free profilin performs as the unique cross-sensitizing agent [60]. Therefore, the absence/presence of ligands does not impact profilin IgE-binding capacity.

Allergen families (lipocalins, nsLTP, and PR-10 proteins) can bind a wide variety of ligands, which can directly interact with the human immune system and/or affect the conformation and stability of allergens [50, 61]. In terms of ligand types, the effects of lipid-binding are discussed in the following section.

Lipocalins have an internal hydrophobic ligand-binding pocket that has a variable structure, allowing ligands of various sizes and shapes to be covalently or non-covalently bound [62]. The ligands can be lipids, steroids, odorants, pheromones, retinoids, and vitamins [63, 64]. Despite exhibiting higher selectivity for ligand-binding, in general, each lipocalin can bind distinct ligands, making it difficult to determine the physiological significance of a specific ligand [63, 64]. Nonetheless, the presence of ligands seems to contribute to the increased allergenicity of lipocalins. PR-10 proteins can bind hydrophobic ligands, such as fatty acids, flavonoids, and steroids [65, 66], as well as plant hormones, such as cytokinins. [67]. IgE-binding and mediator release assays with Bet v 1 variants demonstrated that their allergenicity is unaffected by ligands [68, 69], since it did not enhance the activation of dendritic cells [68]. However, it has also been described that the ligand-binding stabilizes the Bet v 1 structure, resulting in an enhanced melting point as well as resistance towards endo-/lysosomal proteolysis, suggesting that this physicochemical property might be correlated with its high allergenicity. In this sense, humans are exposed to both ligand-bound and free Bet v 1 during the induction phase, revealing a potential key role of the ligand-binding cavity of the allergen associated with increased allergenicity [70].

Structurally, all known α-amylases exhibit a conserved calcium-binding site, with calcium being required to stabilize amylase structure and improve its thermostability [71]. However, the correlation between ligand-binding capacity and the allergenic potential of α-amylases is still quite unknown.

Like other allergens, subtilases also have the capacity to bind calcium as cofactors, contributing to stabilizing their conformational structures, whereas the calcium-binding site is rather flexible and solvent-exposed [72], highlighting the key role of calcium for their biological functions. Yet, no effect on allergenicity has been demonstrated for this allergen family. β-Expansins contain a C-terminal carbohydrate-binding module (CBM) capable of binding carbohydrates, suggesting that their allergenic properties are associated with this structure, as it has been advanced for Lo1 p 1 (rye grass pollen allergen) [73, 74]. Nevertheless, it is still unknown how different ligands (carbohydrates) might affect the IgE-binding capacity of this protein family.

Main findings (Table 1):

- EF-hand members (polcalcins, troponin C), prolamin (α-amylases), and subtilases bind calcium, which is typically fundamental for their functionality.
- Calcium is known to maintain IgE-binding capacity of polcalcins and troponin C, but its role is not known in the case of α-amylases and subtilases.
- Profilins, calycin (lipocalins), and PR-10 proteins bind a wide variety of ligands. Ligands do not affect the allergenicity of profilins, while lipocalins or PR-10 proteins seem to maintain/increase protein allergenicity.
- β-Expansins bind carbohydrates, but their influence on allergenicity is unknown.

### Lipid-Binding

Allergic sensitization is a complex process induced by the allergen and its biological functions, as well as by other small compounds, such as lipids that can bind directly to the allergen as ligands or exist in the allergen source [75]. In food allergens, lipid-binding is known to protect allergens against enzymatic activity and to facilitate their uptake by intestinal epithelial cells, as it has been described for nsLTP and Brazil nut allergen Ber e 1. Additionally, interactions with lipids have also been reported to protect them from proteolysis [2, 3, 75]. Aeroallergens of certain families have also been described as being protected against structural modifications by binding or interacting with lipids.

Within the EF-hand family, FABP are known for their ability to bind hydrophobic ligands such as fatty acids, retinol, retinoic acid, bile salts, and pigments. Der p 13 can bind specific fatty acids and provoke an airway epithelial cell initiation in vitro through TLR2-MyD88-NF-kappaB and MAPK-dependent processes. Der p 13 is known to bind lipids and perform a role in the induction of the house dust mite (HDM)-allergic response through the activation of TLR2 (Toll-like receptor 2) [76]. This highlights a potential correlation between lipid-binding capacity and an increased allergenic potential of the aeroallergens, although no confirmation data is available.

nsLTP can bind miscellaneous lipids [39] with different affinities, allowing to modulate various immunomodulatory activities by affecting their molecular structure and subsequent physicochemical properties or interacting with the immune system [40]. Pru p 3 is described as a food allergen, but it is also an important inhalant sensitizer to respiratory/occupational allergies [31, 77], whose lipid-binding capacity does not alter its allergenicity. Ole e 7 is a nsLTP identified as an aeroallergen, mainly interacting with negatively charged phospholipids and oleic acid, which does not alter its protein structure or its IgE-biding capacity [78].

Main findings (Table 1):

- EF-hand (FABP) and nsLTP can bind specific lipids, maintaining their IgE-binding capacity.

## Conclusions

Some physicochemical properties could be correlated with increased allergenicity of different aeroallergen families, as summarized in Table 1. Abundance, associated with the biological function of most of the studied proteins, seems to be correlated with their increased allergenic potential, although less expressed proteins can also be potent aeroallergens (e.g., tropomyosin, EF-hand proteins). The loss of structural integrity is consensual in reducing allergenicity, but only when conformational epitopes are destroyed and no new epitopic regions are formed (e.g., profilins, lipocalins, PR-10 proteins). However, when oligomerization/aggregation of proteins occurs due to external factors (e.g., heat), increased allergenicity can be observed in oligomers. Similarly to food allergens, the frequency of PTM among aeroallergens is not that high. In fact, only 43% of the protein families herein described present some PTM, with glycosylation being the most common occurrence. Still, not all members of a specific family (e.g. lipocalins) are glycosylated, thus hampering establishing an actual role between glycosylation and protein allergenicity. Ligand- and lipid-binding can also affect protein allergenicity, considering that ligands/lipids can increase the allergenic potency of proteins, although these properties are family/allergen-dependent. It is also important to highlight that the analysis herein reported reflects the authors’ perspective based on the available data/literature. Despite resulting from an extensive literature review, the present work is not a systematic review, meaning that inadvertently, some works might not be included in this analysis.

In summary, despite the available data on aeroallergens, the current knowledge on their structure, function, molecular interactions, and availability is still not enough to draw an overall picture on how physicochemical properties impact their allergenicity. However, the anticipated rising generation of data on aeroallergens owing to advances of the analytical technologies complemented with artificial intelligence will certainly give answers to the big question, “Can physicochemical properties alter the potency of aeroallergens?”, in the near future.

## Data Availability

No datasets were generated or analysed during the current study.
